# Synergism Between *Krameria lappacea* (Dombey) Burdet & B.B.Simpson Extracts and Gentamicin Against Gram-Positive Bacteria

**DOI:** 10.3390/metabo16100699

**Published:** 2026-09-22

**Authors:** Hugo Debelvalet, Francois Gauvin, Régine Janel-Bintz, Marylin Lecsö-Bornet, Joëlle Quetin-Leclercq, Nektarios Aligiannis, Catherine Vonthron-Sénécheau, Raphaël Grougnet, Sabrina Boutefnouchet, Pierre Fechter, Comunidad LickanAntay de Taira, Felix Galleguillos, Sergio Ortiz

**Affiliations:** 1 UMR 7200 Laboratoire d’Innovation Thérapeutique, CNRS, Strasbourg Drug Discovery and Development Institute (IMS), Université de Strasbourg, 67400 Illkirch-Graffenstaden, France; hugo.debelvalet@etu.unistra.fr (H.D.); gauvinfrancois1@gmail.com (F.G.); vonthron@unistra.fr (C.V.-S.); 2 UMR 7242 Biotechnologie et Signalisation Cellulaire (BSC), CNRS Université de Strasbourg, 67400 Illkirch-Graffenstaden, France; janel@unistra.fr (R.J.-B.); p.fechter@unistra.fr (P.F.); 3 Laboratoire Ecosystème Intestinal, Probiotiques, Antibiotiques—EA 4065, Faculté de Pharmacie, Université Paris Cité, 75006 Paris, France; marylin.lecso@parisdescartes.fr; 4 Pharmacognosy Research Group, Louvain Drug Research Institute, UCLouvain, 1000 Brussels, Belgium; joelle.leclercq@uclouvain.be; 5 Department of Pharmacognosy and Natural Products Chemistry, Faculty of Pharmacy, National and Kapodistrian University of Athens, 10431 Athens, Greece; aligiannis@pharm.uoa.gr; 6 UMR CNRS 8038 Team Produits Naturels, Analyses et Synthèse, Faculté de Pharmacie, Université Paris Cité, 75006 Paris, France; raphael.grougnet@u-paris.fr (R.G.); sabrina.boutefnouchet@u-paris.fr (S.B.); 7 Comunidad Indígena Atacameña de Taira, Alto el Loa 1420000, Chile; taira.comunidad@gmail.com; 8 Doctorado en Antropología, Universidad Católica del Norte-Universidad de Tarapacá, San Pedro de Atacama 1410000, Chile; galleguillos.f@gmail.com

**Keywords:** medicinal plant, dereplication, antibacterial synergism, neolignan

## Abstract

**Background**: Combination therapy is increasingly explored as an alternative approach to manage bacterial infections and limit the emergence of antibiotic resistance. Building on traditional medicinal knowledge, this study aimed to investigate the potential synergistic antibacterial effects between *Krameria lappacea* (Dombey) Burdet & B.B.Simpson root extracts and conventional antibiotics. **Methods**: Root extracts were prepared using supercritical fluid extraction with different proportions of ethanol as a co-solvent. Their in vitro antibacterial activity was evaluated against Gram-positive bacteria and their interactions with gentamicin and vancomycin were assessed using an agar dilution-based combination assay. The chemical composition of the extracts was characterized using HRMS/MS-based dereplication and molecular networking. To further investigate the compounds contributing to the observed activity, an MS-guided isolation strategy was applied to an independent *K. lappacea* extract, resulting in the purification of a major neolignan previously detected in the active **KL5** extract, which was subsequently evaluated using broth microdilution assays. **Results**: The less polar extract **KL5**, enriched in neolignans, exhibited the strongest antibacterial activity, with MIC values ranging from 3 to 12 µg/mL against Gram-positive strains. In combination assays, **KL5** exhibited synergistic or additive interactions with gentamicin depending on the bacterial strain, whereas no synergistic effect was observed with vancomycin. The isolated neolignan displayed antibacterial activity and showed an additive interaction with gentamicin against *Staphylococcus* spp. **Conclusions**: These findings support the potential of *K. lappacea* neolignan-rich extracts as antibiotic adjuvants. The activity of the isolated neolignan supports the contribution of this chemical class to the antibacterial properties of **KL5**, while the absence of synergy for the isolated compound suggests that additional constituents and/or multicomponent interactions may contribute to the effect observed for the whole extract.

## 1. Introduction

Although in recent years the development of new antibacterial drugs has become a major public health priority due to the rise in drug resistance, there remain few viable alternatives for accessing novel therapeutic agents [1]. In response to the limited antibacterial pipeline, alternative therapeutic approaches, including non-traditional and indirectly acting antibacterial agents, are increasingly being investigated to address antimicrobial resistance [2]. Among these approaches, antibiotic adjuvants represent a promising strategy to restore or potentiate the activity of existing antibiotics against resistant bacteria [3].

The clinical validity of this strategy is already established for β-lactamase inhibitors, which currently represent the major class of antibiotic adjuvants in clinical use and are administered in combination with β-lactam antibiotics [4]. Beyond these established combinations, alternative adjuvant strategies are currently under preclinical or clinical development, including membrane-permeabilizing agents such as the polymyxin B-derived peptide SPR741, as well as antibiofilm agents and other resistance-modifying compounds [5]. These developments illustrate the diversification of antibiotic-adjuvant strategies beyond conventional β-lactamase inhibition, although their translation into clinical practice remains limited [4,5].

Natural products represent a chemically diverse source of potential antibiotic-potentiating agents, ranging from complex plant extracts to isolated compounds [6]. For example, a polyphenol-rich combination of plant and green propolis extracts showed synergistic interactions with azithromycin and clarithromycin against *Staphylococcus aureus*, as well as with several antibiotics against *Haemophilus influenzae* [7]. Similarly, a hydroethanolic extract of *Origanum vulgare* L. enhanced the in vitro activity of ampicillin and tetracycline against antibiotic-resistant avian pathogenic *Escherichia coli* strains, with synergistic interactions observed for several combinations [8]. At the compound level, berberine has been reported to enhance the activity of several antibiotic classes against multidrug-resistant bacteria, including aminoglycosides [9]. Natural products may potentiate antibiotic activity through diverse mechanisms, including the inhibition of bacterial efflux pumps, interference with resistance determinants, alteration of membrane permeability, and disruption of biofilm formation [10]. Together, these observations highlight the chemical and mechanistic diversity of natural products as potential antibiotic-potentiating agents.

Guided by the search for new antibacterial adjuvants, we investigated the interface between biomedicine and traditional medicine. Traditional medicinal practices, including the use of medicinal plants, remain an important component of health and well-being among LickanAntay communities of northern Chile [11]. Earlier ethnographic studies conducted in the Atacama region documented the concomitant use of traditional remedies and commercial medicines, including antibiotics [12,13,14]. In this traditional pharmacopoeia, the roots of *Krameria lappacea* (Dombey) Burdet & B.B.Simpson (Krameriaceae) have been documented as being prepared as an infusion and traditionally used for urinary disorders, gonorrhea, and pulmonary complaints [12].

In a previous study on the antibacterial potential of LickanAntay traditional medicine, we showed that root extracts of *K. lappacea*, rich in neolignans, displayed potent antibacterial activity with MIC values of 20–40 µg/mL against Gram-positive bacterial strains [15]. In addition to neolignans, *K. lappacea* roots contain condensed tannins and other polyphenolic constituents that are preferentially extracted under more polar conditions [15,16].

Despite the potent antibacterial activity previously reported for *K. lappacea* extracts, their ability to modulate the activity of conventional antibiotics remains largely unexplored. Furthermore, the contribution of individual neolignan constituents to the antibacterial properties of these extracts has not yet been investigated. Therefore, the present study aimed to evaluate the antibacterial interactions between supercritical fluid extracts of *K. lappacea* roots and conventional antibiotics against a panel of Gram-positive bacteria. The extracts were chemically characterized using an HRMS/MS-based molecular networking dereplication approach. In addition, an MS-guided isolation strategy was employed to obtain a major neolignan constituent, which was subsequently evaluated for its antibacterial activity and interaction with gentamicin using broth microdilution assays.

## 2. Materials and Methods

### 2.1. General

Mueller–Hinton agar, lysogeny broth and trypticase soy broth were purchased from BioMérieux (376 Chemin de l’Orme 69280 Marcy-l’Étoile, France). Ethanol (EtOH), dimethyl sulfoxide (DMSO), [3-(4,5-dimethylthiazol-2-yl)-2,5-diphenyl tetrazolium bromide (MTT)], fetal bovine serum (FBS), camptothecin, ofloxacin, gentamicin, and vancomycin were purchased from Sigma-Aldrich (3300 S 2nd St #3306, St. Louis, MO 63118, USA). Silica gel (43–60 nm), acetonitrile, and methanol LC-MS grade were purchased from VWR International (100 Matsonford Road, P.O. Box 6660, Radnor, PA 19087, USA). Ethyl acetate, dichloromethane, and cyclohexane were obtained from CARLO ERBA Reagents (Chau. du Vexin, 27100 Val-de-Reuil, France). Dulbecco’s Modified Eagle Medium (DMEM) and Roswell Park Memorial Institute medium (RPMI) were purchased from Gibco (3175 Staley Rd, Grand Island, NY 14072, USA). ^1^H NMR spectroscopy was performed on Bruker Avance III 400 MHz and 500 MHz spectrometers. The spectra were obtained using CD_3_COCD_3_ as solvent and were analyzed using MestReNova software (version 14.12).

### 2.2. Plant Material

Roots of *K. lappacea* were collected in September 2016 within the LickanAntay Community of Taira, Chile (21°51′36.09′′ S, 68°38′16.67′′ W, 3408 m.a.s.l.). Collection was carried out in accordance with the Chilean National Biodiversity Strategy, and the corresponding Official Material Agreement from the Taira Community was provided and registered at the INIA (Instituto de Investigaciones Agropecuarias, application number 2-2016). Botanical identification was ensured by Prof. Alicia Marticorena (Universidad de Concepción, Chile), and a voucher specimen was deposited in the CONC Herbarium (Universidad de Concepcion, 185883), in the Herbarium of the Laboratoire de Pharmacognosie (Université Paris Cité, MKOSB092015T2B01). An independent commercial batch of roots, purchased from Cailleau Herboristerie (France) in 2021 under the name *Krameria triandra*, was used for the isolation of compound **21**. *K. triandra* Ruiz & Pav. is currently a synonym of *K. lappacea*. A voucher specimen of this material was deposited in the Herbarium of the Laboratoire d’Innovation Thérapeutique (Université de Strasbourg; VON-SO-KL-002).

### 2.3. Extraction

#### 2.3.1. Supercritical Fluid Extraction

The extraction was conducted using a High-Performance Solvent Extraction Lab System, 100 mL (Separex, Champigneulles, France). First, 150 g of dry powdered roots of *K. lappacea* was placed in the stainless-steel extraction cell. Pure CO_2_ was delivered into the extraction basket with a flow of 5.0 kg/h. Ethanol at 5, 10, and 20% was used as co-solvent in the successive extractions to modulate the polarity of the supercritical fluid and obtain extracts with different chemical compositions. The extraction temperature was set at 50 °C and the pressure at 300 bar; these conditions were maintained for 1 h. Extracts were collected after 1 h and stored at −20 °C until further use. The chemical stability of **KL5** during storage was verified by means of HPLC-UV, showing a chromatographic profile consistent with that initially recorded after extraction.

#### 2.3.2. Isolation and Identification of Compound **21**

To obtain sufficient quantities of a major neolignan constituent for biological evaluation, 50 g of powdered roots from the independent commercial *K. lappacea* batch described above were subjected to sequential maceration with 500 mL of dichloromethane. Two consecutive 24 h extractions were performed at room temperature under agitation. The combined dichloromethane extracts were concentrated under reduced pressure to yield the corresponding crude extracts. The extract was fractionated by means of silica gel column chromatography using a cyclohexane/ethyl acetate gradient of increasing polarity. Fractions were pooled according to their TLC profiles, obtaining 13 fractions (F1–F13). LC-MS analysis was then used to identify the fraction containing the ion corresponding to compound **21** previously detected in **KL5**. Fraction 8 was further purified by means of semi-preparative reversed-phase HPLC on a C18 column using a water–acetonitrile gradient containing 0.1% TFA, yielding compound **21** as a purified metabolite.

The structure of compound **21** was elucidated by means of HRMS and NMR analyses (^1^H and ^13^C NMR) and identified as 2-(2,4-dihydroxyphenyl)-5-propenylbenzofuran through comparison with spectroscopic data reported in the literature [17]. The occurrence of compound 21 in **KL5** was further confirmed by matching accurate mass and retention time data and through co-injection with the isolated compound.

### 2.4. Chemical Analysis

#### 2.4.1. HPLC-PDA-HRMS/MS Analysis

Supercritical fluid extracts and the dichloromethane extract used for the MS-guided isolation of compound **21** were analyzed under the chromatographic and mass spectrometric conditions described below. Chromatographic separations were performed on an LC-PDA-HRMS/MS system consisting of a Thermo Accela pump, a PDA detector coupled to a Thermo Scientific LTQ Orbitrap XL mass spectrometer (Thermo Scientific^TM^, Bremen, Germany). The column used was a Phenomenex Luna C18, 250 × 4.6 mm packed with 5 µm particles. Stock solutions were prepared at 10 mg/mL in LC-MS-grade methanol. All analyses were carried out using an injection volume of 20 µL and a flow rate of 0.8 mL/min. PDA detection was performed from 200 to 600 nm. The mobile phase consisted of 0.1% formic acid in water (solvent A) and 0.1% formic acid in acetonitrile (solvent B). Elution was performed in gradient mode: 0–10 min (5% B); 10–40 min (5–100%); 40–45 min (100% B); 45–55 min (100–5% B); 55–60 min (5% B). HRMS analyses were performed in APCI-negative and -positive modes. The inlet conditions for negative mode were: capillary temperature 250 °C; sheath gas flow 25 a.u.; auxiliary gas flow 25 a.u. and sweep gas flow 5 a.u.; APCI vaporizer temperature 400 °C; discharge current of 5 µA; capillary temperature of 250 °C; capillary voltage of −10 V and tube lens voltage of −125 V. For positive mode: capillary temperature 250 °C; sheath gas flow 25 a.u.; auxiliary gas flow 25 a.u. and sweep gas flow 5 a.u.; APCI vaporizer temperature 400 °C; discharge current of 5 µA; capillary temperature of 250 °C; capillary voltage of 21 V and tube lens voltage of 75 V. Data-dependent MS/MS events were performed on the most abundant ions detected in the full-scan MS spectra.

#### 2.4.2. MS Data Treatment

All HRMS .RAW data files were processed using MZmine software suite version 2.5.3. For mass detection at the MS1 level, the noise level was set to 2.0 × 10^5^ for negative mode, which was found to be more informative than positive ionization mode. For MS2 detection, the noise level was set to 1. The ADAP chromatogram builder was used and set to a minimum group size of 5 scans, a minimum group intensity of 2.0 × 10^5^, a minimum highest intensity of 1.0 × 10^5^, and an *m*/*z* tolerance of 10 ppm. The ADAP wavelets algorithm was used for chromatogram deconvolution. The intensity window S/N was used as an S/N estimator with a signal-to-noise ratio set at 6, a minimum feature of 2.0 × 10^5^, a coefficient threshold at 10, a peak duration ranging from 0.02 to 0.6 min, and an RT wavelet range of 0.02 to 0.2 min. Isotopes were detected using the isotope peak grouper with an *m*/*z* tolerance of 8 ppm, an RT tolerance of 0.02 min (absolute), and the maximum charge set at 1, and the most intense isotope was selected as the representative isotope. The aligned list peak was then gap-filled with an RT range of 0.04 min and an *m*/*z* tolerance of 8 ppm. The resulting list was filtered using the peak list row filter option to remove all duplicates and all features without associated MS/MS spectra.

#### 2.4.3. Molecular Network Construction and Dereplication

A molecular network was constructed from the .mgf file exported from MZmine using the online workflow available on the GNPS platform. The precursor ion mass tolerance was set to 0.02 Da and the MS/MS fragment ion tolerance to 0.02 Da. A network was generated in which edges were retained when the cosine score was ≥0.7 with more than three matched fragment ions. Network spectra were then searched against the GNPS spectral libraries using the same filtering parameters. Matches between network and library spectra were retained when the cosine score was ≥0.78. Additional putative annotations of unmatched features were obtained through comparison with MS/MS fragmentation patterns available in the literature. The molecular network was visualized using Cytoscape 3.8.0. Peak area data from the .csv file exported from MZmine were mapped onto the network. Node sizes were set proportionally to the total area of each peak detected in **KL5**, **KL10** and **KL20**.

### 2.5. Biological Evaluations

#### 2.5.1. Bacterial Strains

Bacterial strains were reference strains from French (CIP) or American (ATCC) national collections or clinical strains (E- and N-) responsible for human respiratory, digestive, or urinary infections, such as Gram-positive *Bacillus* sp., *Enterococcus* sp., *Listeria* sp., and *Staphylococcus* sp. and Gram-negative *Salmonella* sp., *Klebsiella* sp., *Pseudomonas* sp., and *Vibrio* sp. This broad panel was selected to provide an initial assessment of the antibacterial spectrum of the extracts across clinically relevant Gram-positive and Gram-negative bacteria. Bacterial strains were cultivated in trypticase soy broth for 18–24 h at 37 °C. Each culture was diluted in sterile distilled water to obtain about 1 × 10^7^ CFU/mL and 1 µL of each (approximately 1 × 10^4^ CFU/spot) was applied to the test medium using a Steers inoculator. Two different panels were used for antibacterial screening. The first panel included 28 Gram-positive and 16 Gram-negative bacterial strains. Based on the activity observed during this initial screening, the second panel focused on 45 bacterial strains belonging to selected Gram-positive genera to further characterize the antibacterial activity of the extracts.

#### 2.5.2. Antibacterial Activity Assay

The in vitro antibacterial activity of the extracts **KL5**, **KL10**, and **KL20** was evaluated using an agar dilution method with ofloxacin as the positive control [11]. Briefly, 250 µL of extract solution in DMSO (4 mg/mL, final concentration of 50 µg/mL) was added to 1.75 mL of sterile distilled water in a Petri dish and Mueller–Hinton agar was added to a final volume of 20 mL and thoroughly mixed. After agar solidification, each bacterial suspension was applied to the test medium using a Steers inoculator. Petri dishes were then incubated for 20 h at 37 °C under aerobic conditions. The corresponding DMSO control was tested under the same conditions and did not affect bacterial growth (1.25% final concentration). Results were assessed visually after incubation. The complete absence of bacterial colonies was considered indicative of growth inhibition. The MIC was defined as the lowest concentration at which no bacterial growth was observed. MIC values for the extracts were determined using seven concentrations (50, 25, 12, 6, 3, 2 and 1 µg/mL). Experiments were performed twice, in triplicate.

For the additional experiments performed with compound **21**, antibacterial activity was evaluated using a broth microdilution assay in sterile 96-well microplates. KL5 was also evaluated using this method under the same experimental conditions to enable direct comparison between the extract and the isolated compound. Serial two-fold dilutions of compound **21** ranging from 100.0 to 0.20 µg/mL and of **KL5** ranging from 250 to 0.49 µg/mL were prepared in lysogeny broth medium. Each well was inoculated with 100 µL of a bacterial suspension adjusted to 5 × 10^5^ CFU/mL and incubated overnight at 37 °C with shaking (200 rpm). Wells containing bacterial inoculum and 4% DMSO served as growth controls, while uninoculated medium served as sterility controls. The MIC was defined as the lowest concentration at which no visible bacterial growth was observed. All experiments were performed in triplicate.

#### 2.5.3. Fractional Inhibitory Concentration Index (FICI) Determination

The interactions between KL5 and gentamicin or vancomycin were evaluated using an agar dilution-based combination assay. Briefly, 125 µL of a solution of **KL5** in DMSO was combined in a Petri dish with 125 µL of a solution (gentamicin or vancomycin) prepared in sterile water. The concentrations of **KL5** used were 50, 25, 12, 6, 3, 1.5, 0.75, and 0.375 µg/mL, while the corresponding antibiotic concentrations were 2, 1, 0.5, 0.25, 0.125, and 0.0625 µg/mL. Mueller–Hinton agar was then added to each Petri dish to a final volume of 20 mL and homogenized. After agar solidification, bacterial suspensions were inoculated using a Steers inoculator as described above for the agar dilution assay. The fractional inhibitory concentration (FIC) of each agent was calculated by dividing its MIC in combination by its MIC when tested alone. The fractional inhibitory concentration index (FICI) was calculated as the sum of the two FIC values.

In contrast, the interaction between compound **21** and gentamicin was evaluated using a broth microdilution checkerboard assay in sterile 96-well microplates. Compound **21** was tested at concentrations ranging from 6.000 to 0.500 µg/mL, while gentamicin was tested at concentrations ranging from 4.000 to 0.125 µg/mL. Following inoculation with bacterial suspensions (5 × 10^5^ CFU/mL), plates were incubated overnight at 37 °C with shaking (200 rpm).

An FICI value <0.5 was interpreted as synergistic, 0.5–1 as additive, >1–2 as showing no interaction, and >2 as antagonistic [18]. Experiments were performed twice in triplicate.

#### 2.5.4. Cytotoxic Activity Assay

Murine macrophage cell line J774 (ECACC 91051511), human lung fibroblast cell line WI-38 (ATCC CCL-75), and human hepatocellular carcinoma cell line HepG2 (ATCC HB-8065) were obtained from LGC Standards (Molsheim, France). J774 cells were cultured in RPMI 1640 medium, whereas WI-38 and HepG2 cells were cultured in DMEM. Both media were supplemented with 10% FBS and penicillin–streptomycin (100 U/mL) and maintained at 37 °C under 5% CO_2_. Cells were seeded in a 96-well plate at a density of 5 × 10^3^ cells/well in 180 µL per well of the respective culture medium. After 24 h, cells were treated with 20 µL of different concentrations of each extract (20 mg/mL stock solution, subsequently diluted in the respective culture medium to a final concentration ranging from 100 to 0.04 µg/mL) and incubated for an additional 72 h. After incubation, the medium was removed and 100 µL of MTT solution (300 µg/mL in the respective culture medium) was added to each well. Plates were incubated for 45 min, after which the MTT solution was replaced with 100 µL of DMSO per well. Absorbance was measured at 570 nm, using 620 nm as the reference wavelength, with a SpectraMax spectrophotometer (Molecular Devices, Berkshire, UK). Camptothecin was used as a positive control. Experiments were performed at least three times in triplicate.

## 3. Results

Collected roots of *K. lappacea* were successively extracted using supercritical fluid extraction with CO_2_ as the primary solvent and ethanol at 5% (**KL5**), 10% (**KL10**), and 20% (**KL20**) as a co-solvent. The extraction yields were 6.8%, 5.3%, and 2.6%, respectively. Chemical characterization of the supercritical fluid extracts was performed using a molecular networking-based dereplication approach combining HPLC-PDA-HRMS/MS analysis with the Global Natural Products Social Molecular Networking (GNPS) platform, as previously described [19]. This approach enables HRMS/MS-based putative annotation through comparison with the extensive collection of annotated natural product MS/MS spectra available in the GNPS spectral libraries [20].

The resulting molecular networks provide a graphical representation of the ions detected via HPLC-PDA-HRMS/MS, organized into distinct molecular clusters according to their spectral similarities. Networks obtained for **KL5**, **KL10**, and **KL20** revealed mainly polyphenolic compounds, grouped into clusters corresponding to neolignans (Figure 1, Clusters B and H), methoxy-catechins (Cluster C), phenylethyl-hexosides (Cluster D), and flavonolignan derivatives (Cluster G).

After a polyol isomer (**1**), an eluted compound was putatively identified as a dihydroxyphenylethyl-hexosyl-pentoside isomer (**2**), which produced a formate molecular ion [M+HCOO^−^]^−^ at *m*/*z* 493, fragmenting to the corresponding [M−H]^−^ ion at *m*/*z* 447 and a fragment at *m*/*z* 151, consistent with the neutral loss of the hexosyl-pentosyl moiety (Table 1). Phenylethyl-hexosyl-hexoside (**4**) was identified with a formate molecular ion [M+HCOO^−^]^−^ at *m*/*z* 491 and a fragment at *m*/*z* 163, assigned to C_6_H_11_O_5_^−^ and consistent with cleavage of the interglycosidic bond. Similar fragmentation was observed for compound **7**, identified as a phenylethyl-hexosyl-pentoside, with a formate molecular ion [M+HCOO^−^]^−^ at *m*/*z* 461 and a fragment at *m*/*z* 283, corresponding to the neutral loss of C_6_H_10_O_6_ from the formate adduct.

The flavonolignans putatively annotated included two cinchonain isomers (**5** and **11**) and larixinol or one of its isomers (**14**). The cinchonain isomers **5** and **11** displayed a deprotonated ion [M−H]^−^ at *m*/*z* 451, with fragment ions at *m*/*z* 341 [M–C_6_H_6_O_2_–H]^−^, *m*/*z* 299 [M–C_6_H_6_O_2_–CH=CH–OH–H]^−^, and *m*/*z* 217 [M–2C_6_H_6_O_2_–CH–H]^−^, corresponding to the loss of two dihydrobenzene rings and of a methylene group, characteristic of cinchonain structures [21,22]. Larixinol (or one of its isomers, **14**) was detected as a deprotonated ion [M−H]^−^ at *m*/*z* 541, with fragment ions at *m*/*z* 415 [M–C_6_H_6_O_3_–H]^−^, *m*/*z* 497 [M–CO_2_–H]^−^, and *m*/*z* 309 [M–C_6_H_6_O_3_–C_7_H_6_O–H]^−^, corresponding respectively to the loss of a trihydroxybenzene moiety, CO_2_, and a trihydroxybenzene together with a methylene-substituted benzene moiety, characteristic of larixinol-type structures [23].

Three catechin derivatives were putatively annotated: catechin (**8**, [M−H]^−^ at *m*/*z* 289), a trimethoxycatechin (**10**, [M−H]^−^ at *m*/*z* 331), and a dimethoxycatechin isomer (**12**, [M−H]^−^ at *m*/*z* 317). Compounds 10 and 12 both displayed a fragment at *m*/*z* 207 corresponding to the loss of a hydroxy-methoxybenzene ring. Further fragment ions were observed at *m*/*z* 123 [M–C_11_H_12_O_4_–H]^−^ for **10** and *m*/*z* 109 [M–C_11_H_12_O_4_–H]^−^ for **12** [24].

Regarding the neolignan derivatives, five compounds were putatively annotated: **16** ([M−H]^−^ at *m*/*z* 283, C_17_H_16_O_4_), **17** ([M−H]^−^ at *m*/*z* 313, C_18_H_18_O_5_), **19** ([M−H]^−^ at *m*/*z* 297, C_18_H_18_O_4_), **21** ([M−H]^−^ at *m*/*z* 265, C_17_H_14_O_4_), and **23** ([M−H]^−^ at *m*/*z* 277, C_18_H_14_O_3_). Their molecular formulas were consistent with those of neolignans previously isolated from the *Krameria* genus [17,25,26]. Compounds **16** (2-(2,4-dihydroxyphenyl)-5-(3-hydroxypropyl)benzofuran), **21** (2-(2,4-dihydroxyphenyl)-5-propenylbenzofuran), and **23** (3-formyl-2-(4′-hydroxyphenyl)-5-propylbenzofuran) showed similar fragmentation patterns, including the loss of a CO group yielding fragment ions at *m*/*z* 255, 237, and 249, respectively. This fragmentation is consistent with literature reports describing decarbonylation of the furan ring followed by ring contraction in related neolignans [27,28].

In addition, the neolignan **17** (2-(2-methoxy-3,4-dihydroxyphenyl)-5-(3-hydroxypropyl)benzofuran or its isomer 2-(2-methoxy-4,6-dihydroxyphenyl)-5-(3-hydroxypropyl)benzofuran) and the neolignan **19** (5-(3-hydroxypropyl)-2-(2-methoxy-4-hydroxyphenyl)benzofuran or its positional isomer) displayed fragment ions at *m*/*z* 298 and 282 respectively, corresponding to the loss of a methyl group from the methoxy substituent.

Two other constituents were also putatively annotated: chlorogenic acid (**6**) and a hydroxy-tri-methoxy-flavan isomer (**18**).

To the best of our knowledge, ten phenolic derivatives (**2**, **4**, **5**, **6**, **7**, **10**, **11**, **12**, **14**, and **18**) are reported here for the first time in *K. lappacea* through molecular networking analysis.

**Table 1:** Putative identification of chemical constituents present in *K. lappacea* extracts (**KL5**, **KL10** and **KL20**) through comparison of the MS/MS data (negative mode, APCI). * First report in *K. lappacea*.

Code	RT [min]	UV [λ_max_]	*m*/*z*	MS Major Ion(s)	Molecular Formula	Fragmentation [*m*/*z*]	ΔmDa	Identification	Previously Isolated from *Krameria* Genus
**1**	3.58	n.d.	197.0674 151.0616	[M+HCOO^−^]^−^ [M−H]^−^	C_5_H_12_O_5_	71.01430	0.95	Polyol isomer	
						101.0249			
						133.0511			
**2**	17.11	282	493.1564	[M+HCOO^−^]^−^	C_19_H_28_O_12_	447.1508	0.55	Dihydroxyphenylethyl-hexosyl-pentoside *	
						151.0613			
**3**	17.35	271	413.1653	[M−H]^−^	C_16_H_30_O_12_	367.1607	−0.60	n.i.	
						221.1033			
**4**	17.62	277	491.1776	[M+HCOO^−^]^−^	C_20_H_30_O_11_	307.1037	0.51	Phenylethyl-hexosyl-hexoside *	
						445.1715			
						163.0615			
**5**	18.54	280	451.1027	[M−H]^−^	C_24_H_20_O_9_	341.0668	−0.21	Cinchonain isomer I *	
						299.0566			
						217.0145			
						189.0198			
**6**	19.09	288, 327	353.0883	[M−H]^−^	C_16_H_18_O_9_	191.0565	2.95	Chlorogenic acid	
**7**	19.30	280	461.1700	[M+HCOO^−^]^−^	C_19_H_28_O_10_	415.1611	0.68	Phenylethyl-hexosyl-pentoside *	
						283.1196			
**8**	19.89	282	289.0724	[M−H]^−^	C_15_H_14_O_6_	245.0822	1.19	Catechin I	
						205.0510			
						179.0356			
**9**	20.32	278	597.2205	[M+HCOO^−^]^−^	C_27_H_36_O_12_	551.2137	1.54	n.i.	
						435.1084			
**10**	21.08	280	331.1200	[M−H]^−^	C_18_H_20_O_6_	207.0666	1.84	Trimethoxy-catechin *	
						179.0720			
						151.0769			
						123.0458			
**11**	21.21	281	451.1057	[M−H]^−^	C_24_H_20_O_9_	341.0669	2.79	Cinchonain isomer II *	
						217.0145			
						299.0566			
**12**	21.35	280	317.1035	[M−H]^−^	C_17_H_18_O_6_	207.0664	0.99	Dimethoxy-catechin *	
						299.1291			
						109.0299			
						149.0611			
**13**	22.81	278	299.0931	[M−H]^−^	C_17_H_16_O_5_	281.0822	1.15	n.i.	
						237.0923			
**14**	23.14	277, 317	541.1147 587.1215	[M−H]^−^ [M+HCOO^−^]^−^	C_30_H_22_O_10_	415.0829	1.23	Larixinol isomer *	
						497.1254			
						307.0623			
						309.0414			
						455.1147			
						267.0308			
**15**	23.57	n.d.	301.1082	[M−H]^−^	C_17_H_18_O_5_	191.0718	0.60	n.i.	
						109.0300			
**16**	24.83	281, 316	283.0981	[M−H]^−^	C_17_H_16_O_4_	265.0878	1.07	2-(2,4-dihydroxyphenyl)-5-(3-hydroxypropyl)benzofuran	[25]
						255.1032			
						238.0638			
**17**	25.08	283, 317	313.1082	[M−H]^−^	C_18_H_18_O_5_	298.0850	0.60	2-(2-methoxy-3,4-dihydroxyphenyl)-5-(3-hydroxypropyl)benzofuran or 2-(2-methoxy-4,6-dihydroxyphenyl)-5-(3-hydroxypropyl)benzofuran	[25]
**18**	25.79	284, 314	315.1246	[M−H]^−^	C_18_H_20_O_5_	191.0719	1.35	Hydroxy-trimethoxy-flavan isomer	
						123.0458			
						108.0222			
**19**	26.66	293, 317	297.1140	[M−H]^−^	C_18_H_18_O_4_	282.0903	1.32	5-(3-hydroxylpropyl)-2-(2-methoxy-4-hydroxyphenyl)benzofuran or 5-(3-hydroxylpropyl)-2-(2-hydroxy-4-methoxyphenyl)benzofuran	[25]
**20**	28.25	283	579.2394	[M−H]^−^	C_36_H_36_O_7_	535.2132	1.12	n.i.	
						561.2282			
**21**	30.02	289, 319	265.0874	[M−H]^−^	C_17_H_14_O_4_	237.0925	0.93	2-(2,4-dihydroxyphenyl)-5-propenylbenzofuran	[17,25,26]
						221.0969			
**22**	30.77	n.d.	563.2437	[M−H]^−^	C_36_H_36_O_6_	225.0919	0.34	n.i.	
						337.1446			
						323.1293			
						469.2024			
**23**	31.08	261, 304	277.0876	[M−H]^−^	C_18_H_14_O_3_	249.0922	1.13	3-formyl-2-4′-hydroxyphenyl-5-propenylbenzofuran	[25]
**24**	32.51	n.d.	527.1887	[M−H]^−^	C_36_H_36_O_5_	265.0875	2.85	n.i.	
						277.0872			
						249.0923			
**25**	33.23	n.d.	531.2189	[M−H]^−^	C_35_H_32_O_5_	265.0871	1.75	n.i.	
						305.1186			
**26**	36.67	n.d.	525.2078	[M−H]^−^	C_36_H_30_O_4_	294.0898	1.22	n.i.	
						262.0999			
						261.0922			
						233.0971			

To compare the relative distribution of the different polyphenol families in the three extracts, peak area data were integrated into the molecular network obtained for *K. lappacea* extracts. The node size in the network was adjusted to be proportional to the peak area of each ionized compound (Figure 1). Based on this semi-quantitative representation, neolignans (clusters B and H) and methoxy-catechins (cluster C) showed higher relative peak areas in the less polar extract, **KL5**. In contrast, flavonolignans (cluster G) showed higher relative peak areas in the most polar extract, **KL20**. These trends are consistent with previous reports, describing flavonolignans as being more prevalent in extracts obtained with polar solvents and neolignans as predominant in less polar extracts [15,29,30].

The antibacterial activity of the extracts was evaluated using the agar dilution method against two panels of bacterial strains. The first panel was designed to determine the general spectrum of activity against Gram-negative and Gram-positive bacteria, while the second comprised an extended panel of selected Gram-positive bacteria, including reference strains and clinical isolates, to determine the minimum inhibitory concentrations (MIC) of **KL5**, **KL10**, and **KL20**.

In the initial screening, the extracts inhibited only Gram-positive bacteria at a final concentration of 50 µg/mL. Consequently, MIC determination was carried out exclusively against Gram-positive bacteria, including reference and clinical strains belonging to the genera *Bacillus*, *Corynebacterium*, *Enterococcus*, *Staphylococcus,* and *Listeria*.

The strongest antibacterial activity was observed for **KL5**, with MIC values ranging from 3 to 12 µg/mL (Table 2) against the Gram-positive panel. **KL10** and **KL20** were less active, with MIC values of 12–50 µg/mL and 50 µg/mL or higher, respectively. The bacterial strains most sensitive to **KL5** belonged to the genus *Enterococcus*, with four of six strains (*E. casselliflavus* CIP 103.018, *E. faecalis* CIP 103.214 and CIP 104.676, and *E. faecium* CIP 107.387) exhibiting MIC values of 3 µg/mL.

For the reference strains of *Staphylococcus* spp., *Listeria* spp., and *Bacillus* spp., **KL5** inhibited growth at concentrations between 3 and 6 µg/mL, as it did for the clinical isolates *S. aureus*, *S. caprae*, *S. hominis*, *S. intermedius*, *S. saprophyticus* (N270 and N315), and *S. warneri*. The strains less sensitive to **KL5** (MIC = 12 µg/mL) included *Corynebacterium minutissimum* CIP 100652T, *E. faecium* CIP 103.014, *E. hirae* CIP 5855, and the clinical isolates *S. capitis* N1154, *S. sciuri* N993, and *S. saprophyticus* N238 and N854.

**KL20** was the least active extract, inhibiting growth only at 50 µg/mL against *B. cereus* CIP 6624 and N839, *C. striatum* N1129, *E. faecium* CIP 103.014, *S. aureus* ATCC 25923 and E372, *S. hominis* N1143, *S. saprophyticus* N238, *S. warneri* N1243, and *L. monocytogenes* N836.

As a positive control, ofloxacin showed MIC values of 0.12–2 µg/mL against *Bacillus* spp., *Corynebacterium* spp., *E. faecalis*, *S. aureus* strains (except for the clinical isolated strains E372 and E385, with MIC values of 16 and 64 µg/mL, respectively), *S. lugdunensis* ATCC 43809, *S. saprophyticus* strains, *S. sciuri* N993, *S. warneri* N1243, and *L. monocytogenes* strains. It was less active against *E. casselliflavus* CIP 103.018 and *E. faecium* CIP 107.387 (MIC = 64 and 32 µg/mL, respectively) and against the clinical isolates *S. hominis* N1143 and *S. intermedius* N987, which exhibited reduced susceptibility to ofloxacin (MIC = 128 and 16 µg/mL, respectively).

Using the same agar dilution-based combination assay described above, we evaluated the interaction between the most active antibacterial extract **KL5** and two commercial antibiotics, gentamicin and vancomycin. These antibiotics belong to distinct antibacterial classes and were selected to explore whether the interaction with **KL5** differed according to the antibiotic tested. The **KL5**/gentamicin combination exhibited synergistic effects against both reference and clinical isolates belonging to *Bacillus*, *Listeria*, and *Staphylococcus* strains, with FICI values ranging from 0.27 to 0.48 (Table 3). In contrast, additive effects (FICI values of 0.5–1) were observed against *S. aureus* ATCC 9144, *S. aureus* E373, *S. hominis* N1143, *S. intermedius* N987, *L. monocytogenes* CIP 103.575, *Corynebacterium* spp., and *Enterococcus* spp. Thus, the interaction between **KL5** and gentamicin was strain-dependent, ranging from additive to synergistic among the tested Gram-positive bacteria. The **KL5**/vancomycin combination showed indifferent effects, with FICI values between 1.0 and 2.0 (Table 3).

To further investigate the contribution of individual constituents to the antibacterial activity of **KL5**, a major neolignan previously detected in the extract (compound **21**) was isolated from an independent *K. lappacea* root extract using an MS-guided purification strategy. The identity of compound **21** was established by means of HRMS and NMR analyses and confirmed through comparison with literature data. Its occurrence in **KL5** was further verified by matching accurate mass and retention time data, as well as through co-injection experiments.

As the biological evaluation of compound **21** was performed using broth microdilution, **KL5** was also evaluated using this method. Under these conditions, **KL5** exhibited MIC values ranging from 125 to 250 µg/mL, substantially higher than those obtained using the agar dilution method (3–12 µg/mL). Compound **21** displayed antibacterial activity against all three *S. epidermidis* ATCC12228, *S. aureus* RN6390, and USA300 strains, with MIC values of 3.00, 12.5, and 6.25 µg/mL, respectively (Table 4).

To evaluate its potential contribution to the antibiotic-potentiating effect observed for **KL5**, the interaction between compound **21** and gentamicin was evaluated using a broth microdilution checkerboard assay. Compound **21** exhibited additive interactions against all three strains, with FICI values of 0.83, 0.68, and 0.70 for ATCC12228, RN6390, and USA300, respectively (Table 4). No synergistic interactions (FICI < 0.5) were observed for the purified compound under the tested conditions.

To provide a preliminary assessment of the cytotoxicity of the extracts toward mammalian cells, the cytotoxic effects of the *K. lappacea* supercritical fluid extracts were evaluated. Three different cell lines were tested: J774 murine macrophages, WI-38 human lung fibroblasts, and HepG2 human hepatocellular carcinoma cells. Cytotoxicity was measured using the MTT reduction assay, and the results were expressed as IC_50_ values, with camptothecin used as a positive control. The effects on cell viability are summarized in Table 5. The lowest IC_50_ values for all three extracts were observed in the J774 cell line, with values of 22.03 ± 1.59, 21.49 ± 3.21, and 23.46 ± 2.15 µg/mL for **KL5**, **KL10**, and **KL20**, respectively. Conversely, the highest IC_50_ values were recorded against the HepG2 cell line, at 53.82 ± 1.74, 46.36 ± 1.69, and 41.74 ± 2.51 µg/mL for **KL5**, **KL10**, and **KL20**, respectively.

## 4. Discussion

The detection of several neolignans (**16**, **17**, **19**, **21**, and **23**) in the supercritical fluid extracts **KL5**, **KL10**, and **KL20** is consistent with previous reports on the chemical constituents of *K. lappacea*. Compounds **19**, **21**, and **23** have also been reported in *K. cystisoides*, *K. interior*, *K. grayi*, and *K. parvifolia* [17,26,29,31]. Catechin and its derivatives (**8**, **10**, and **12**) are well-known constituents of the *Krameria* genus and have been isolated from the roots of *K. lappacea* and *K. pauciflora* [32,33]. Condensed tannins, which are typically produced by *Krameria* species, were not detected in **KL5**, **KL10**, or **KL20** under our HPLC-PDA-HRMS/MS conditions. Previous work has shown that these tannins can be extracted using supercritical fluid procedures with more polar co-solvents and higher proportions of methanol (up to 40% for 2 h) [34], which may explain their absence under our extraction conditions. This was further supported by the 4-dimethylaminocinnamaldehyde assay, which did not detect significant amounts of condensed tannins in our extracts.

In earlier studies, ethanolic extracts of *K. lappacea* roots exhibited MIC values of 64 µg/mL against *S. aureus* ATCC 6538 and ten methicillin-resistant *S. aureus* strains using a microdilution method [16]. The same extract inhibited biofilm formation by *S. aureus* ATCC 6538 at 256 µg/mL but was not able to disrupt preformed biofilms. In our previous work on the antimicrobial activity of *K. lappacea*, the ethyl acetate extract inhibited bacterial growth with MIC values of 20 µg/mL against both reference and clinical *Staphylococcus* strains and 40 µg/mL against *Enterococcus* strains. In contrast, the methanolic extract showed moderate activity, with MIC values of 80–100 µg/mL [15].

In the present study, the less polar extract **KL5** exhibited the most significant in vitro antibacterial activity against Gram-positive bacteria, showing lower MIC values than the ethyl acetate extract previously obtained by means of pressurized solvent extraction and evaluated using the same agar dilution methodology. This observation suggests that supercritical fluid extraction using 5% ethanol as a co-solvent yielded an extract with greater antibacterial activity under these experimental conditions. In our previous bioassay-guided fractionation, the ethyl acetate extract yielded the neolignan (+)-conocarpan, which showed MIC values of 0.5–2 µg/mL against Gram-positive strains [15]. However, (+)-conocarpan was not detected in our current extracts by HPLC-PDA-HRMS/MS.

In previous studies, the antibacterial activity of neolignans **16**, **17**, **21**, and **23** was evaluated via disk diffusion [25]. Compounds **16** and **17** produced inhibition zones of 11–14 mm against *B. subtilis* and *B. cereus* (200 µg per disk), while compound **21** displayed the strongest activity, with inhibition zones of 17 mm against both *Bacillus* strains and **16** mm against *E. coli*. Compound **23** was inactive under the same conditions. Antimicrobial activity has also been reported for structurally related benzofuran neolignans from other plant species. Recently, (+)-conocarpan, eupomatenoid-5, and eupomatenoid-6 isolated from *Piper rivinoides* were shown to inhibit *Mycobacterium tuberculosis*, with MIC_50_ values of 1.46, 2.23, and 1.04 µg/mL, respectively, against the H37Rv strain [35]. Together, these observations indicate that lignan-derived compounds occurring in *Krameria* and structurally related benzofuran neolignans can exhibit antimicrobial activity, although this activity appears to depend strongly on their chemical structure and the microorganism considered. Flavonolignans, such as cinchonain isomers, have also shown moderate antibacterial activity, with MIC values of 310–620 µg/mL against *B. cereus*, *S. aureus*, *E. coli* and *P. aeruginosa* [36]. Although some isolated lignans and flavolignans have been reported to exhibit weak activity against Gram-negative bacteria, our preliminary screening of extracts included both Gram-positive and Gram-negative strains and revealed antibacterial activity only against Gram-positive bacteria. Consequently, all subsequent antibacterial and combination assays with the supercritical fluid extracts were performed using Gram-positive strains.

Previous studies have demonstrated strong synergism between gentamicin and various plant extracts against Gram-positive bacteria. For example, gentamicin combined with an ethanolic extract of *Glycyrrhiza glabra* (licorice) yielded an FICI of 0.19 against *Listeria monocytogenes* ATCC 19115 [37]. Similarly, gentamicin in combination with dichloromethane extracts of *Plectranthus ornatus*, *Salvia officinalis,* or *Senna macranthera* showed FICI values between 0.25 and 0.375 against *S. aureus* strains 3993 and 4125 [38]. Thymol exhibited synergistic interactions with gentamicin against *S. aureus*, with FICI values of 0.375–0.50 and a 75% reduction in the gentamicin MIC [39]. Eugenol also showed synergy with gentamicin against *L. monocytogenes* (FICI = 0.50), reducing the MIC of both compounds by 75% [40]. In our study, synergistic interactions between **KL5** and gentamicin were similarly observed for a subset of the tested Gram-positive strains, while additive interactions occurred for the remaining strains. In contrast, no synergistic interaction was observed between **KL5** and vancomycin. Although this difference may indicate that the antibiotic-potentiating effect of **KL5** depends on the antibiotic considered, testing a broader range of antibiotic classes will be required before any relationship with specific mechanisms of antibiotic action can be established.

Although the mechanisms underlying these synergistic interactions remain largely unresolved, several hypotheses have been proposed for plant-derived metabolites. Epigallocatechin gallate, for example, restored aztreonam susceptibility in multidrug-resistant *P. aeruginosa* (FICI = 0.02–0.5), possibly by increasing bacterial membrane permeability and antibiotic uptake, as suggested by ethidium bromide accumulation assays [41]. Likewise, the *C*-glycosyl flavone vitexin exhibited strong synergy with azithromycin and gentamicin (FICI = 0.182 and 0.078, respectively) against *P. aeruginosa* MTCC 2488 while also reducing biofilm formation. Molecular docking and in vitro assays suggested that vitexin interacts with quorum sensing-associated proteins, including LuxR, LasA and LasI [42]. Together, these studies indicate that plant secondary metabolites may enhance antibiotic efficacy through multiple complementary mechanisms. These observations prompted us to investigate whether a major constituent of **KL5** could contribute to the observed antibiotic-potentiating effect. Therefore, a major neolignan detected in **KL5** was isolated from a new *K. lappacea* root extract and subsequently evaluated for its antibacterial activity and interaction with gentamicin.

The biological evaluation of compound **21** was performed using broth microdilution, whereas the initial antibacterial and combination assays of **KL5** were conducted using agar dilution. When **KL5** was additionally evaluated by means of broth microdilution, MIC values of 125–250 µg/mL were obtained, compared with 3–12 µg/mL in the agar dilution assay. This marked difference highlights the influence of experimental conditions on the apparent antibacterial activity of the extract. Differences between agar- and broth-based assays may be particularly relevant when evaluating lipophilic natural products, for which limited aqueous solubility and dispersion can affect their behavior in liquid culture media. Such methodological constraints have been particularly documented for essential oils and other hydrophobic natural products [43,44]. Given the relatively lipophilic composition of **KL5**, similar physicochemical factors could potentially influence the availability of its constituents in broth; however, whether this applies to neolignan-rich extracts remains to be established. Moreover, because the two assays were performed using different bacterial strain panels in addition to different assay formats, the origin of this discrepancy cannot be attributed to a single experimental factor. Consequently, quantitative comparisons between results obtained using the two methodologies should be interpreted with caution.

Interestingly, although compound **21** exhibited antibacterial activity and an additive interaction with gentamicin, it did not reproduce the synergistic effect observed for the crude **KL5** extract. The antibacterial activity of compound **21**, together with its confirmed occurrence in **KL5**, supports the hypothesis that this neolignan may contribute to the antibacterial properties of the extract. However, because the combination assays for **KL5** and compound **21** were performed using different experimental formats and bacterial strain panels, their FICI values cannot be directly compared. The absence of synergy for compound **21** under the tested conditions therefore does not establish the mechanism underlying the synergistic behavior of **KL5** but suggests that compound **21** alone is insufficient to reproduce it. Other constituents, including additional neolignans detected in the molecular network, and/or multicomponent interactions may contribute to the effect observed for the whole extract.

This observation is consistent with the concept that the biological activity of complex botanical extracts often results from the contribution of multiple constituents acting through complementary or overlapping mechanisms. In this context, plant-derived secondary metabolites have been reported to potentiate antibiotic activity through diverse modes of action, including alteration of bacterial membrane integrity, inhibition of efflux systems, interference with resistance mechanisms, and disruption of biofilm formation [45,46,47]. Such effects may increase intracellular antibiotic accumulation or sensitize bacterial cells to antimicrobial agents. Although the present study was not designed to investigate these mechanisms, the fact that compound **21** alone did not reproduce the synergistic interaction observed for **KL5** raises the hypothesis that additional constituents or multicomponent interactions may be involved in the potentiation of gentamicin activity. The isolation and biological evaluation of compound **21** nevertheless provide additional support for the potential contribution of neolignans in the antibacterial activity of *K. lappacea*. Further phytochemical investigations aimed at isolating additional constituents from **KL5**, together with mechanistic studies of their interactions with conventional antibiotics, will be necessary to better understand the molecular basis of the synergistic effects observed in this study.

In the present study, the three supercritical fluid extracts showed IC_50_ values of approximately 21–23 µg/mL against J774 macrophages and higher values against WI-38 and HepG2 cells. Although these IC_50_ values were generally higher than the antibacterial MIC values obtained for **KL5** by means of agar dilution, the relatively limited difference between antibacterial and cytotoxic concentrations, particularly for J774 cells, warrants caution. Moreover, MIC and IC_50_ represent different biological endpoints and were determined using distinct experimental systems and exposure conditions; therefore, a direct quantitative comparison cannot establish a defined selectivity window. The present cytotoxicity results should consequently be considered a preliminary assessment of mammalian cell toxicity. Further studies will be required to characterize the selectivity and safety of **KL5** before its potential use as an antibiotic adjuvant can be established.

## 5. Conclusions

This study revealed marked chemical and biological differences among *K. lappacea* supercritical fluid extracts. Neolignans were predominant among the detected constituents of the less polar extract **KL5**, whereas flavolignans and other polar metabolites were more abundant in the most polar extract. Ten phenolic derivatives were reported for the first time in this species. Consistent with our previous findings on ethyl acetate extracts, **KL5** exhibited the strongest antibacterial activity against Gram-positive bacteria and displayed synergistic interactions with gentamicin against a subset of the tested strains, whereas no synergistic interaction was observed with vancomycin. The isolation and biological evaluation of a major neolignan constituent (compound **21**) support the potential contribution of this class of metabolites to the antibacterial activity of *K. lappacea*. However, compound **21** showed only additive interactions with gentamicin under the tested conditions, suggesting that this compound alone is insufficient to reproduce the synergistic effect observed for **KL5** and that additional constituents and/or multicomponent interactions may be involved. These findings emphasize the importance of investigating botanical extracts as multicomponent systems rather than attributing their biological activity solely to their major constituents. Given the cytotoxicity observed toward mammalian cells and the methodological limitations identified in comparing the different antibacterial assays, the potential of **KL5** as an antibiotic adjuvant should be considered preliminary. Nevertheless, these findings support further phytochemical, mechanistic, and safety investigations of neolignan-rich *K. lappacea* extracts to identify the constituents and interactions underlying their antibiotic-potentiating effects.

## Figures and Tables

**Figure 1:**
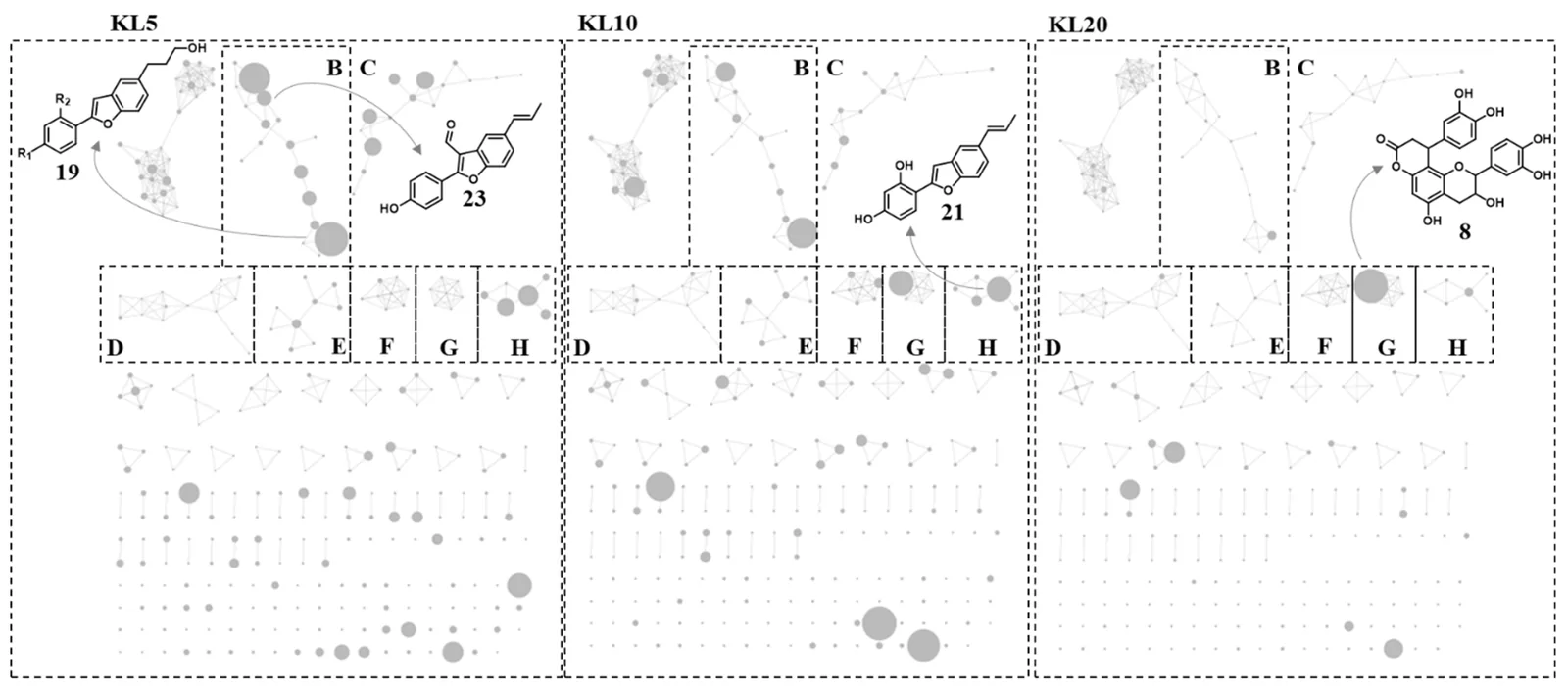
Molecular network of *K. lappacea* extracts (**KL5**, **KL10**, and **KL20**). Clusters corresponding to neolignans (B, E, F and H), methoxy-catechins (C), phenylethyl-hexosides (D), and flavonolignan derivatives (G).

**Table 2:** In vitro antibacterial activity of *K. lappacea* supercritical fluid extracts and the control antibiotic.

Reference	Bacteria Species	MIC Values of Extracts and Reference (µg/mL)			
		KL5	KL10	KL20	Ofloxacin
CIP 6624	*Bacillus cereus*	6	25	50	0.5
N551	*Bacillus cereus*	6	25	>50	0.12
N839	*Bacillus cereus*	3	12	50	0.5
N1060	*Bacillus cereus*	6	25	>50	0.5
N1139	*Bacillus cereus*	6	25	>50	0.12
CIP 100652T	*Corynebacterium minutissimum*	12	50	>50	0.12
N1129	*Corynebacterium striatum*	3	12	50	0.5
CIP 103.018	*Enterococcus casselliflavus*	3	50	>50	64
CIP 103.214	*Enterococcus faecalis*	3	12	>50	2
CIP 104 676	*Enterococcus faecalis*	3	12	>50	1
CIP 103.014	*Enterococcus faecium*	12	25	50	8
CIP 107.387	*Enterococcus faecium*	3	12	25	32
CIP 105 985	*Enterococcus gallinarum*	3	>50	>50	4
CIP 5855	*Enterococcus hirae*	12	25	>50	2
ATCC 25.923	*Staphylococcus aureus*	6	25	50	0.5
ATCC 29213	*Staphylococcus aureus*	6	25	>50	0.5
ATCC 6538P	*Staphylococcus aureus*	6	25	>50	0.25
ATCC 9144	*Staphylococcus aureus*	6	25	>50	0.25
CIP 57.10	*Staphylococcus aureus*	6	25	>50	0.25
E372	*Staphylococcus aureus*	6	50	50	16
E373	*Staphylococcus aureus*	6	25	>50	1
E378	*Staphylococcus aureus*	6	25	>50	4
E384	*Staphylococcus aureus*	3	12	>50	0.25
E385	*Staphylococcus aureus*	3	50	>50	64
E387	*Staphylococcus aureus*	6	25	>50	0.5
E410	*Staphylococcus aureus*	6	25	>50	0.5
E415	*Staphylococcus aureus*	6	25	>50	0.5
N1154	*Staphylococcus capitis*	12	50	>50	8
N1039	*Staphylococcus caprae*	6	25	>50	0.5
CIP 53.124	*Staphylococcus epidermidis*	6	25	>50	0.5
CIP 81.56	*Staphylococcus haemolyticus*	6	25	>50	0.25
N1143	*Staphylococcus hominis*	6	50	50	128
N987	*Staphylococcus intermedius*	6	25	>50	16
ATCC 43.809	*Staphylococcus lugdunensis*	3	12	>50	0.25
CIP 76125	*Staphylococcus saprophyticus*	6	25	>50	1
N238	*Staphylococcus saprophyticus*	12	50	50	1
N270	*Staphylococcus saprophyticus*	6	25	>50	1
N315	*Staphylococcus saprophyticus*	6	50	>50	1
N854	*Staphylococcus saprophyticus*	12	50	>50	1
N993	*Staphylococcus sciuri*	12	50	>50	1
N1243	*Staphylococcus warneri*	3	12	50	0.5
CIP 103.575	*Listeria monocytogenes*	6	25	>50	2
N783	*Listeria monocytogenes*	6	25	>50	2
N836	*Listeria monocytogenes*	6	25	50	2
N851	*Listeria monocytogenes*	6	25	>50	1

**Table 3:** Synergistic effects of the combination of **KL5** with commercial antibiotics.

Fractional Inhibitory Concentration Index (FICI) Between KL5 and Commercial Antibiotics					
Reference	Bacterial Species	KL5 + Gentamicin	MIC Gentamicin	KL5 + Vancomycin	MIC Vancomycin
CIP 6624	*Bacillus cereus*	0.27	2.0	1.0	0.5
N551	*Bacillus cereus*	0.48	1.0	1.0	0.5
N1060	*Bacillus cereus*	0.48	0.5	1.5	0.25
N1139	*Bacillus cereus*	0.48	1.0	1.0	0.5
ATCC 6538P	*Staphylococcus aureus*	0.48	0.5	1.5	1.0
ATCC 9144	*Staphylococcus aureus*	0.62	0.5	1.0	1.0
CIP 57.10	*Staphylococcus aureus*	0.48	0.5	1.5	1.0
E373	*Staphylococcus aureus*	0.53	0.5	1.5	1.0
E387	*Staphylococcus aureus*	0.27	0.5	1.5	0.5
E410	*Staphylococcus aureus*	0.27	0.5	1.5	0.5
E415	*Staphylococcus aureus*	0.27	0.5	2.0	0.5
N1143	*Staphylococcus hominis*	0.53	2.0	1.5	0.5
N987	*Staphylococcus intermedius*	0.53	2.0	1.0	1.0
CIP 103.575	*Listeria monocytogenes*	0.53	1.0	1.0	0.5
N783	*Listeria monocytogenes*	0.27	1.0	1.5	0.5
N836	*Listeria monocytogenes*	0.27	1.0	1.0	0.5
N851	*Listeria monocytogenes*	0.27	0.5	1.5	0.5

**Table 4:** In vitro antibacterial activity (MIC) of compound **21** and synergistic evaluation with gentamicin measured by means of broth-dilution methodology.

Bacterial Species	21 (µg/mL)	Gentamicin (µg/mL)	FICI
*Staphylococcus epidermidis ATCC*12228	3.00	1.00	0.83
*Staphylococcus aureus* RN6390	12.50	2.50	0.68
*Staphylococcus aureus* USA300	6.25	4.00	0.70

**Table 5:** In vitro cytotoxic activity of *K. lappacea* supercritical fluid extracts and positive control camptothecin.

IC_50_ (µg/mL) Values of *K. lappacea* Extracts and Positive Control Against Selected Cell Lines				
Cell line	KL5	KL10	KL20	Camptothecin
J744	22.0 ± 1.5	21.4 ± 3.2	23.4 ± 2.1	0.01 ± 0.01
WI-38	28.4 ± 3.2	27.4 ± 2.4	31.2 ± 3.1	0.02 ± 0.01
HepG2	53.8 ± 1.7	46.3 ± 1.6	41.7 ± 2.5	0.02 ± 0.01

## Data Availability

The original contributions presented in this study are included in the article. Further inquiries can be directed to the corresponding author.
